# The role of lung ultrasound in COVID-19 disease

**DOI:** 10.1186/s13244-021-01013-6

**Published:** 2021-06-19

**Authors:** European Society of Radiology (ESR), Dirk-André Clevert, Paul S. Sidhu, Adrian Lim, Caroline Ewertsen, Vladimir Mitkov, Maciej Piskunowicz, Paolo Ricci, Núria Bargallo, Adrian P. Brady

**Affiliations:** Am Gestade 1, Vienna, Austria

**Keywords:** COVID-19, Lung ultrasonography, Pneumonia, Point-of-care ultrasound (POCUS), Artefacts

## Abstract

This statement summarises basic settings in lung ultrasonography and best practice recommendations for lung ultrasonography in COVID-19, representing the agreed consensus of experts from the Ultrasound Subcommittee of the European Society of Radiology (ESR). Standard lung settings and artefacts in lung ultrasonography are explained for education and training, equipment settings, documentation and self-protection.

## Keypoints

- Chest multislice-CT is still regarded as the gold-standard imaging technique for thoracic evaluation.
- Lung ultrasonography can show typical pattern for interstitial pneumonia.
- COVID-19 lesions mainly involve the peripheral pulmonary zones, which makes this disease accessible for pulmonary ultrasonography evaluation.
- Lung ultrasonography can make a substantial contribution, as it allows direct bedside examination of the lung and pleural space.
- Lung ultrasonography in experienced hands can give results that are comparable to chest MS-CT and superior to standard chest radiography for assessment of pneumonia and/or adult respiratory distress syndrome (ARDS).

## Introduction

According to the Center for Systems Science and Engineering (CSSE) at Johns Hopkins University (JHU), Coronavirus COVID-19 disease prevalence has increased globally day-to-day since early 2020, passing a total of more than 111 million infections worldwide in mid-February 2021, and more than 2.45 million deaths worldwide by this date. At the time of writing, the number of proven cases continues to rise, particularly in the USA, Russia, South America and Europe [1]. On 12 March 2020, the World Health Organization labelled the outbreak as a pandemic infection with a previously unknown type of coronavirus named SARS-CoV-2 [2]. The infection, formally named COVID-19 (Coronavirus disease 2019) by the WHO on February 11th. 2020, is usually fairly mild, but causes severe pneumonia in a minority of infected patients, potentially developing into respiratory failure and death due to bilateral pulmonary involvement [3]. The extensive and rapid spread of the infection, with the need of advanced health care support, has made this pandemic a serious health emergency.

Spreading mainly through the droplet route, the virus predominantly causes mild symptoms, the most common being temperature (80%), dry cough (56%), fatigue (22%) and muscle pain (7%); less common signs include a painful throat, a runny nose, diarrhoea, haemoptysis and chills [4]. A life-threatening difficulty of SARS-CoV-2 contamination is an acute respiratory distress syndrome (ARDS) [5], which happens more frequently in older adults, persons with immune disorders and co-morbidities. Severe forms of the infection (respiratory failure, ARDS, sepsis and septic shock) require management in the intensive care unit and, sometimes, mechanical ventilatory support [4].

Due to the continual development of their clinical condition, critically ill patients need thoracic imaging frequently [6]. Thoracic ultrasonography can make a substantial contribution, as it allows direct bedside examination of the lung and pleural space [7]. Patients with COVID-19 show typical CT patterns, such as diffuse bilateral interstitial pneumonia, with irregular, patchy lesions distributed mainly in the periphery of the lung [8–15].

Multislice-CT remains the gold standard imaging method for thoracic evaluation [16]. As pulmonary irregularities may develop before clinical appearances and confirmation of viral infection by polymerase chain reaction (PCR), some have suggested primary lung CT for screening suspected patients [17], regardless of limited sensitivity. However, lung MS-CT examinations expose patients to ionising radiation, and should be reserved for specific purposes, for instance circumstances establishing the baseline status of severely ill patients, confirmation of pulmonary embolism and evaluation of mediastinal pathologies [18–20]. The standard of care in the ICU is still bedside chest X-ray (CXR). Nevertheless, CXR has significant methodological limitations and is often of low accuracy in identifying disease [21]. Additionally, it is important to consider radiation protection issues [22]. Chest CT has limitations in unstable ICU patients with hypoxemia and hemodynamic failure. Additionally, there are risks of causing harm when transporting the patient, and the high contagiousness of SARS-CoV- 2 could induce spread of the infection in the hospital [16, 23].

Lung ultrasonography in experienced hands can give results comparable to chest CT and superior to standard chest radiography for evaluation of pneumonia and/or adult respiratory distress syndrome (ARDS), with the additional benefit of ease of use, repeatability, absence of radiation exposure, and low cost [17, 24]. The appropriate practice of ultrasonography could decrease CXR and CT use in patients in the ICU [7].

## Fundamentals of lung ultrasonography examinations

Thoracic and lung ultrasonography have increased in importance over the past decade [25–30], particularly in the setting of point of care ultrasonography (POCUS) [31].

A lung ultrasonography examination was first executed more than half century ago [32, 33]. Excepting echocardiography and obstetrical use, ultrasonography has predominantly been a tool used by radiologists and the lung wasn´t considered suitable for this imaging technology historically [34–36]. Nonetheless, since 1991, intensivists have been using ultrasonography in a variety of settings, including in vascular access, to detect free fluid in body cavities and, to a lesser extent, in lung assessment [37].

Severe dyspnoea is a common presenting initial sign in the emergency room and the ICU. The variety of thinkable differential diagnoses is extensive; after history-taking, physical examination and determination of the vital parameters, prompt bedside emergency ultrasonography can be helpful. Focused pulmonary ultrasonography is a significant component of emergency ultrasonography beside dedicated ultrasonography of the abdomen and heart. While recommendations exist for elective chest ultrasonography [38, 39] and point of care pulmonary ultrasonography [25], emergency pulmonary ultrasonography has not yet been extensively adopted in everyday practice [40]. In experienced hands, pulmonary ultrasonography shows brilliant diagnostic accurateness in identifying pleural effusion, pneumothorax, pulmonary venous congestion and consolidation, compared to clinical examination and CXR [21, 41, 42].

In the diagnosis of lung pathologies, ultrasonography artefacts arising from the chest wall and pleural surface can provide valuable information and may correlate with the existing lung pathophysiology. There are two major ultrasonographic artefact patterns that a clinician may detect: “A-lines” and “B-lines” in rare cases even “C-lines” are visible [43].

**Fig. 1:**
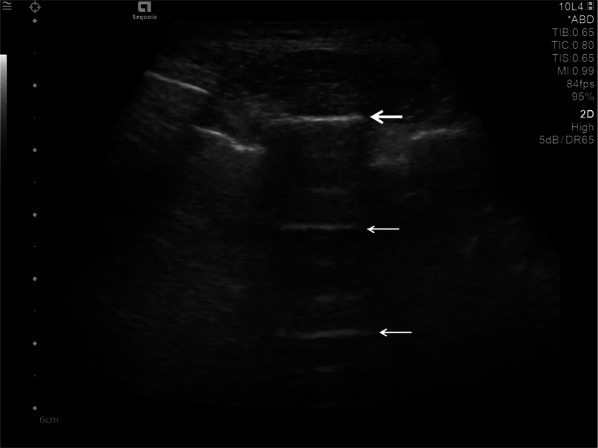
Ultrasonography detected A-lines using an abdominal probe. A-lines (white arrows) appears as bright horizontal lines deep to the pleural line (bold white arrow)

A-lines are reverberation artefacts triggered by oscillating tissue with an air interface, causing the ultrasonography waves to be reflected strongly and to reverberate [22, 44]. Among the probe and lung surface, the ultrasonography waves bounce back and forth. A-lines are parallel horizontal repetition lines of the pleural surface, appearing deeper on the display screen (Fig. 1). Due to the fact that this is a classic reverberation artefact, the distance from the skin to the pleural surface equals the distance from the pleural line to the first A-line, the first A-line to the second A-line, and so forth. The A-profile is shaped by intact (“dry”) lung parenchyma containing air when it is combined with normal lung sliding. If sliding is absent, it is intensely suggestive of a pneumothorax [45].

**Fig. 2:**
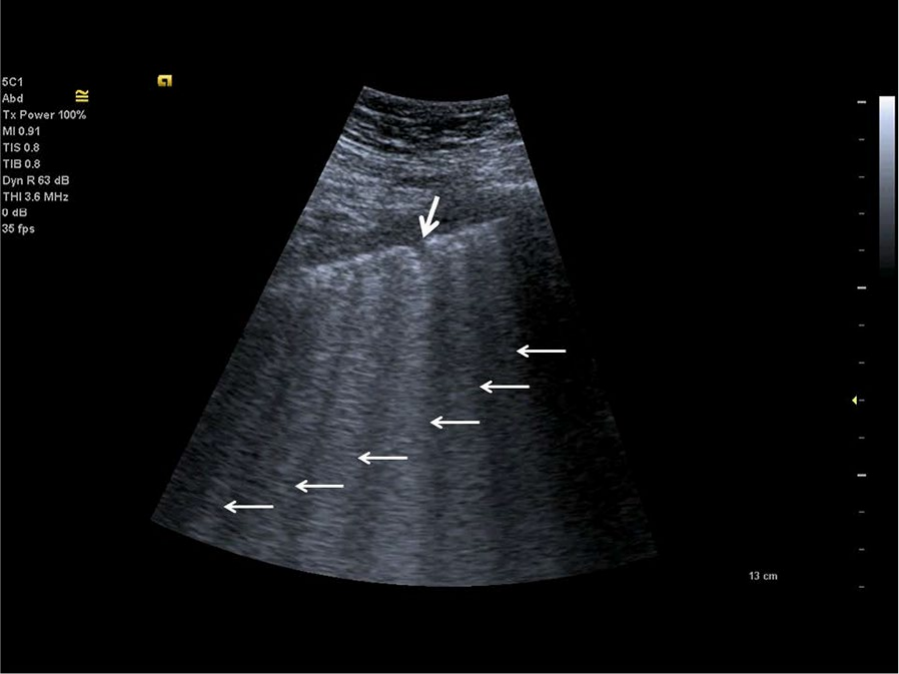
Lung ultrasonography, in comparison to Fig. 3, there is an increased density of B-lines (white arrows) indicating an interstitial syndrome of the lung with irregular, discontinuous pleural line with a small subpleural consolidation (bold white arrow)

In 1982, Ziskin et al. first described the “comet-tail” ultrasonographic sign, seen when an intrahepatic shotgun pellet was detected to generate an artefact like to what is seen in celestial comets [45]. On lung ultrasonography, usual comet-tail artifacts originate at the pleura but fade before reaching the edge of the screen. According to the international consensus conference, B-lines are spreading to the bottom of the screen without fading, not only pronounced [25, 48]. B-lines seen as vertical hyperechoic artefacts originating from the pleura or areas of consolidation [46]. B-lines indicate an accumulation of fluid in the pulmonary interstitial space or alveoli. The presence of several B-lines is related with pulmonary oedema, of cardiogenic, noncardiogenic (acute respiratory distress syndrome) or mixed origin. Additionally multiple B-lines are reported in pulmonary fibrosis, and pneumonia [25, 48]. B-lines arise when sound waves pass through the superficial soft tissues and cross the pleural line, encountering a mixture of air and water [23]. The anatomic and physical basis of B-lines is not known with certainty at this time [25]. One or two B-lines may be normal, but when they increase in number or spread in one zone, they are a sign of severe pulmonary interstitial oedema [39, 47] (Fig. 2).

**Fig. 3:**
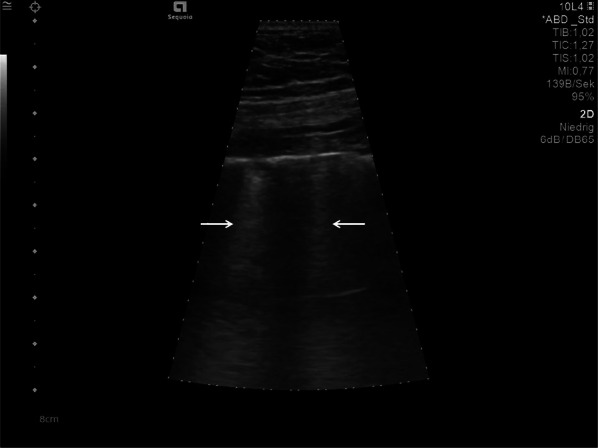
Examination of the lung using a curved array probe, detecting single C-lines (white arrows), they come out of tiny subpleural consolidations

C-lines are small anterior lung consolidations suggest pneumonia; they are 18 times more often than pulmonary embolism [34] (Fig. 3).

## Transducer and system settings

For preoperative and postoperative transthoracic ultrasonography, most conventional US systems are suitable, using the "real-time B-mode" technique. There is a broad selection of different ultrasonography transducers which may be used for lung ultrasonography (LUS) [39, 48]. Each transducer has specific advantages and limits; transducer choice depends on numerous factors, principally patient anatomy, size and age and the depth and nature of the visualised structures [23].

Published studies on lung ultrasonography have used a variety of transducers, including high-frequency linear, low-frequency curvilinear and low-frequency sector transducers. In most of the cases, the use of convex and linear transducer will be sufficient [47]. Low-frequency probes will offer greater depth penetration, but will sacrifice some image quality. High-frequency probes will offer better resolution to observe lesions in the pleural line, but will sacrifice depth penetration [47, 48]. The performance and interpretation of lung ultrasonography is not probe-specific [48].

### Convex transducers

The most suitable transducer for bedside lung ultrasonography (BLUE) will be low-frequency convex transducers, because they can visualise the deep postero-lateral structures and can reveal consolidation and pleural effusion [23, 45]. In patient with COVID-19 disease, the whole lungs have to be scan because the process especially at the beginning can be visible in multiple places in order not to miss interstitial changes.

### Linear transducers

High-frequency linear transducers, with more-shallow penetration, may be superior for identifying pneumothorax and examining the superficial anterior structures. They are suitable for children and thin adults [39].

### Phased-array and microconvex transducers

Specialised phased-array and microconvex transducers can be used to detect a broad variety of abnormalities, such as consolidation and pleural effusion [47].

### System settings

In order to evaluate vessels and the vascularisation of pathological findings, basic ultrasonography units should be equipped with pulsed and colour Doppler and M-mode [49].

The diagnostic value of B-mode ultrasonography can be enhanced by using the dynamic M-mode. In the M-mode setting, a solitary vertical line of the ultrasonography image is selected. The detected ultrasonography signals of this line are displayed over time in a separate diagram. By using this M-mode setting, tissue movements will be represented as curves. Structures which are not moving appear as a horizontal line. By using the M-mode technique, the system displays a representation of tissue motion over time during the lung examination [44].

In order to improve B-mode quality, most ultrasonography manufacturers use Compound Imaging and Harmonic Imaging. By disabling these modes, comet-tail artefacts or B-lines will be displayed more clearly [39, 44].

Standard settings such as focal zone, image depth and overall gain should be adjusted to improve visualisation of the pleural line [50].

## Self-protection, ultrasound machine and transducer cleaning

### Self-protection

Due to the extended exposure time of ultrasonography operators scanning COVID-19 patients, the risk of coronavirus transmission will be increased. Therefore adequate personal protective equipment should be worn while examination COVID-19 positive patients or patients with a high risk of being COVID-19 positive. According to Piscaglia et al., personal protective equipment should include FFP2 (N95) or FFP3 faces masks, gloves, disposable caps, shoe covers, protective glasses, goggles or face barriers in order to protect operators from potentially infectious patients, and also to protect patients from contracting COVID-19 from asymptomatic healthcare personal [39, 47, 51, 52].

### Ultrasound system

Most ultrasonography systems are not designed for cleaning with liquid disinfectants. Extended disinfection with liquid disinfectant solutions will almost invariably damage the controls, knobs and keypad. To resolve this problem, the US machine should be covered entirely with transparent, thin, disposable nylon/plastic bags. This may interfere with cooling of the ultrasonography system, and potential overheating should be monitored. An additional option is to only cover the screen and the main keyboard/controls [53, 54]. Unfortunately, if no protective cover is used, it is difficult to be certain that every part of the scanner has been disinfected after use. Some recently developed ultrasonography scanners have adapted to these considerations, increasing machine resistance to disinfectants and making it easier to clean and disinfect the main body of the equipment [51].

### Transducer cleaning

Under standard ultrasonography examination circumstances a cleaning tissue to wipe the probes might be adequate [55, 56]. Nevertheless, due to the increase in hazardous transmittable infections such as SARS-CoV-2 or methicillin-resistant staphylococcus, most manufacturers and scientific societies tend to suggest procedures such as immersion of the transducer in a disinfectant solution for 1–5 min, or, alternatively, in a stronger disinfectant solution for at least 30 s [51]. Unfortunately, these procedures are complex and time-consuming, and therefore acceptance of these suggestions may be limited [15, 53]. Additionally, the immersion of probes in potent disinfectant could damage some probes after as few as 50–100 disinfecting cycles, making such procedures impracticable. In order to avoid most of these problems, disinfecting procedures should be of minimal complexity, while preserving patient and probe safety [51]. The use of a thin and disposable nylon/plastic cover for the transducer can minimise these problems [23, 47]. Advanced cleaning solutions must be discussed with local infection control and the vendors supplying the machine in order to avoid any damage to the system and transducer [53, 54].

## Basic lung examination

Lung ultrasonography is used in emergency and ICU patients in recumbent (ventral thorax) and—depending on the clinical situation—in a sitting position (dorsal thorax). In order to cover the complete lung, the examination should be performed in a systematic manner that investigates the entire anterolateral and posterior lung surfaces bilaterally; if necessary, selective regional lung ultrasonography can be performed with a patient-focused abbreviated approach [39].

According to Lu et al., each hemithorax should be divided into six regions [57]: anterior superior, anterior inferior, lateral superior, lateral inferior, posterior superior and posterior inferior. These areas should be marked as R1–R6 and L1–L6, respectively, a standard pictorial proforma could be used to fill so that response in follow-up US studies can be accurately documented [23, 39, 47]

Usually, transthoracic scanning windows, using the intercostal spaces, will be used for the examination of the lung and pleura [40]. In most standard examinations, a convex array transducer will be used and should be placed at right angles to the ribs so that two adjacent ribs are captured. This setting allows the lung to slide, i.e. the movement of the visceral pleura can be reliably identified and distinguished from the anterior rib artefact. In order to cover the complete lung, this technique should be used for each intercostal space of the upper and lower parts of the anterior, lateral and posterior regions as well of the left and right chest wall [58]. If subsequently focusing on suspected subpleural lesions, a linear array transducer may improve the resolution of superficial structures [39].

## Ultrasonography findings in patient with COVID-lung disease

Ultrasonography can be useful in addition to CT and CXR, or on its own, for the primary diagnosis and follow-up of COVID-19 disease [22]. COVID-19 pneumonia is more likely than non-COVID-19 pneumonia to have a peripheral distribution [59].

**Fig. 4:**
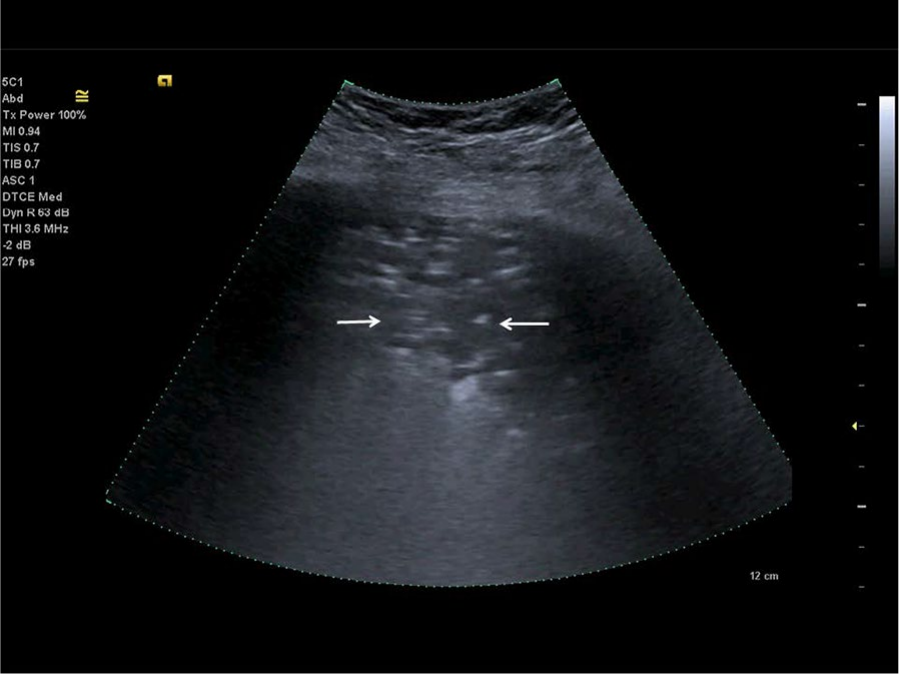
Follow-up examination in the Intensive Care Unit. Detection of consolidations with visible air bronchograms (white arrows)

The morphology and variations of subpleural lesions, changes of air and water content in consolidated peri-pulmonary tissues and air bronchograms, can be detected, using the low-frequency capability of curved transducers [23] (Fig. 4).

**Fig. 5:**
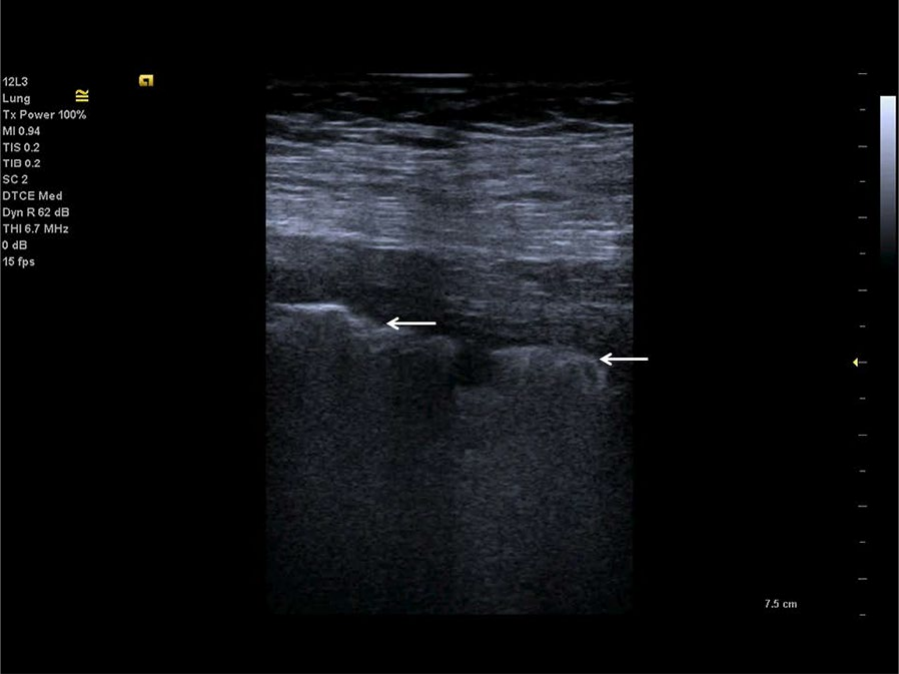
Lung ultrasonography using a linear probe. Discontinuous pleura (white arrows) with tiny round hypoechoic consolidations

The findings and treatment of central lung diseases is currently limited in lung ultrasonography, due to the attenuation of sound waves by normal lung and bone tissues. The diagnosis of lung ultrasonography abnormalities relies on the artefacts created by peri-pulmonary lesions [60, 61]. An abnormal ratio of air and water content in alveoli and interstitial tissues created these artefacts. In lung ultrasonography of COVID-19 disease, we typically find thickening of the pleural line with pleural line abnormality. Additionally, the pleural line may appear discontinuous (interrupted) [17, 62] (Fig. 5).

The presence of B-lines artefacts (> 3 B-lines per intercostal space) will vary among focal, multifocal and confluent patterns of involvement [23]. The extent of consolidation may change in different patterns, from multifocal, small, subpleural consolidations up to non-translobar and translobar involvement, with occasional air bronchograms [23, 63].

**Fig. 6:**
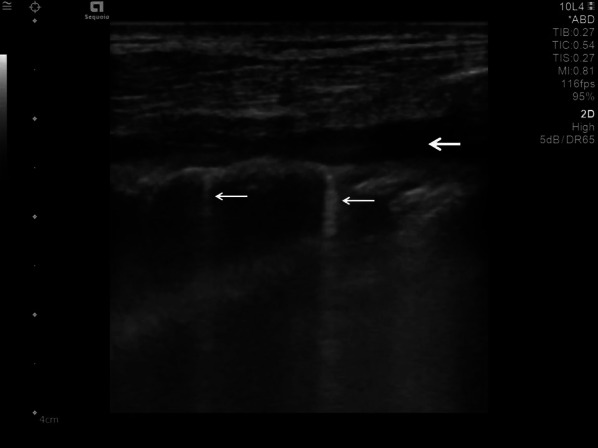
Lung ultrasonography using a linear probe. B-lines (white arrows) with irregular pleura and a small pleural effusion (bold white arrow)

**Fig. 7:**
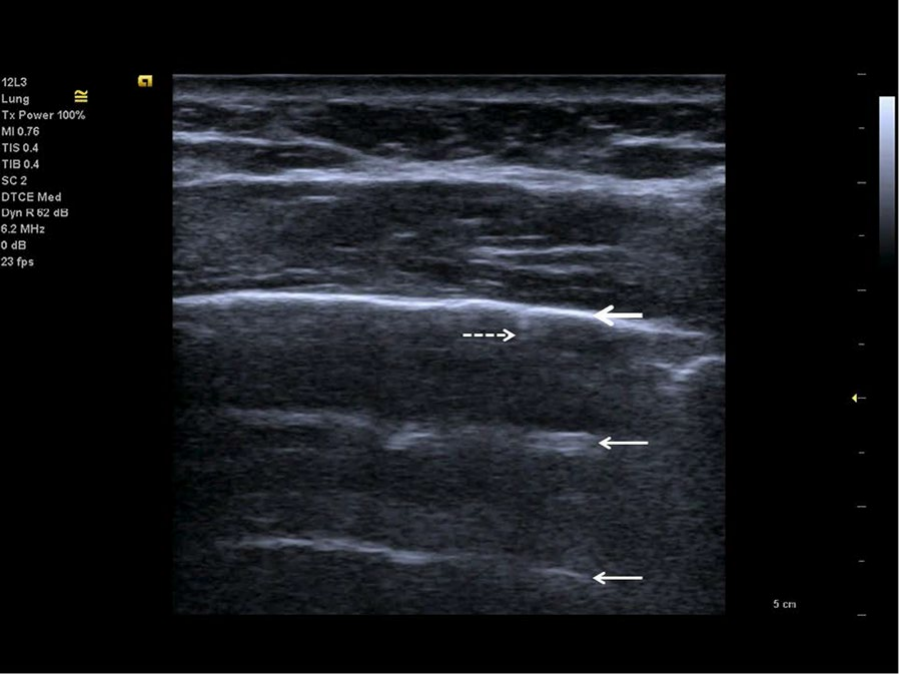
Follow-up of an ICU patient with COVID-19 pneumonia after mechanical ventilation. An indirect sign of recovery is the appearance of A-lines (white arrows) through the recovery phase. No major pleural fragmentation (bold white arrow) is seen, but a mirror effect is visible (left white arrow)

In COVID-19 lung disease, pleural effusions are uncommon; patients are generally more critically ill if pleural effusions are seen (Fig. 6). An indirect sign of recovery is the presence of ultrasonography A-lines through the recovery phase [23, 39, 47, 64] (Fig. 7).

## Conclusions

Lung ultrasonography has substantial use in management of COVID-19 pneumonia in the ICU, due to its non-invasive assessment and dynamic observation of lung lesions [39, 64]. Lung ultrasonography can show typical pattern for interstitial pneumonia; in COVID-19, it is mainly involving the peripheral pulmonary zones [57]. Lung ultrasonography can be used for quick valuation of the severity of SARS-CoV-2 pneumonia, to track the evolution of disease during follow-up and to monitor lung recruitment manoeuvres. Additionally, ultrasonography can track the response to prone-position ventilation and the controlling of extracorporeal membrane therapy [17]. Major advantages are the safety of lung ultrasonography, its repeatability and low cost [39, 51]. With enlarged use of bedside ultrasonography in the ICU, patients can be protected from needless radiation and therapy delays. CT may be reserved for follow-up in cases where lung ultrasonography is unable to answer the clinical question. The transport of high-risk patients to CT examinations, with its associated hazards, can be decreased by increasing use of lung ultrasonography.

## Data Availability

Image data are stored in the local PACS at the University of Munich.

## Abbreviations

ARDS: Adult respiratory distress syndrome
BLUE: Bedside lung ultrasonography
CSSE: Center for Systems Science and Engineering
CXR: Chest X-ray
ESR: European Society of Radiology
PCR: Polymerase chain reaction
POCUS: Point-of-care ultrasound

## References

[1] John Hopkins University & Medicine. Coronavirus Resource Center. https://coronavirus.jhu.edu/map.html. Last accessed 4 March 2021.

[2] Cucinotta D, Vanelli M (2020). WHO declares COVID-19 a pandemic. Acta Biomed.

[3] Zhu N, Zhang D, Wang W et al (2020) A novel coronavirus from patients with pneumonia in China. N Engl J Med 382:727–733 10.1056/NEJMoa2001017 PMC7092803 31978945

[4] Wujtewicz M, Dylczyk-Sommer A, Aszkiełowicz A (2020). COVID-19—what should anaethesiologists and intensivists know about it?. Anaesthesiol Intensive Ther.

[5] Soldati G, Giannasi G, Smargiassi A, Inchingolo R, Demi L (2020). Contrast-enhanced ultrasound in patients with COVID-19: pneumonia, acute respiratory distress syndrome, or something else?. J Ultrasound Med.

[6] Brogi E, Bignami E, Sidoti A (2017). Could the use of bedside lung ultrasound reduce the number of chest x-rays in the intensive care unit?. Cardiovasc Ultrasound.

[7] Oks M, Cleven KL, Cardenas-Garcia J (2014). The effect of point-of-care ultrasonography on imaging studies in the medical ICU: a comparative study. Chest.

[8] Wu J, Wu X, Zeng W (2020). (2020) Chest CT findings in patients with corona virus disease 2019 and its relationship with clinical features. Invest Radiol.

[9] Zhao W, Zhong Z, Xie X, Yu Q, Liu J (2020) Relation between chest CT findings and clinical conditions of coronavirus disease (COVID-19) pneumonia: a multicenter study. AJR Am J Roentgenol 214(5):1072–1077 10.2214/AJR.20.22976 32125873

[10] Zhou S, Wang Y, Zhu T, Xia L (2020) CT features of coronavirus disease 2019 (COVID-19) pneumonia in 62 patients in Wuhan, China. AJR Am J Roentgenol 214(6):1287–1294 10.2214/AJR.20.22975 32134681

[11] Xiong Y, Sun D, Liu Y (2020). Clinical and high-resolution CT features of the COVID-19 infection: comparison of the initial and follow-up changes. Invest Radiol.

[12] Ye Z, Zhang Y, Wang Y, Huang Z, Song B (2020). Chest CT manifestations of new coronavirus disease 2019 (COVID-19): a pictorial review. Eur Radiol.

[13] Liu M, Zeng W, Wen Y (2020). COVID-19 pneumonia: CT findings of 122 patients and differentiation from influenza pneumonia. Eur Radiol.

[14] Niu R, Ye S, Li Y (2021). Chest CT features associated with the clinical characteristics of patients with COVID-19 pneumonia. Ann Med.

[15] Revel M-P, Parkar A, Prosch H (2020). COVID-19 patients and the Radiology Department—advice from the European Society of Radiology (ESR) and the European Society of Thoracic Imaging (ESTI). Eur Radiol.

[16] Dunn MJ, Gwinnutt CL, Gray AJ (2007). Critical care in the emergency department: patient transfer. Emerg Med J.

[17] Peng QY, Wang XT, Zhang LN, Chinese Critical Care Ultrasound Study Group (CCUSG) (2020). Findings of lung ultrasonography of novel corona virus pneumonia during the 2019–2020 epidemic. Intensive Care Med.

[18] Tecce PM, Fishman EK, Kuhlman JE (1994). CT evaluation of the anterior mediastinum: spectrum of disease. Radiographics.

[19] Brenner DJ, Hall EJ (2007). Computed tomography—an increasing source of radiation exposure. N Engl J Med.

[20] Tapson VF (2012). Advances in the diagnosis and treatment of acute pulmonary embolism. F100 Med Rep.

[21] Lichtenstein D, Goldstein I, Mourgeon E (2004). Comparative diagnostic performances of auscultation, chest radiography, and lung ultrasonography in acute respiratory distress syndrome. Anesthesiology.

[22] Gargani L, Picano E (2015). The risk of cumulative radiation exposure in chest imaging and the advantage of bedside ultrasound. Crit Ultrasound J.

[23] Bard RL (2021) Image-guided management of COVID-19 lung disease. Springer Nature

[24] Mayo PH, Copetti R, Feller-Kopman D (2019). Thoracic ultrasonography: a narrative review. Intensive Care Med.

[25] Volpicelli G, Elbarbary M, Blaivas M (2012). International evidence-based recommendations for point-of-care lung ultrasound. Intensive Care Med.

[26] Volpicelli G (2013). Lung sonography. J Ultrasound Med.

[27] Dietrich CF, Gebhard Mathis G, Cui XW (2015). Ultrasound of the pleurae and lungs. Ultrasound Med Biol.

[28] Mathis G (2010). Why look for artifacts alone when the original is visible?. Chest.

[29] Gargani L, Pang PS, Frassi F (2015). Persistent pulmonary congestion before discharge predicts rehospitalization in heart failure: a lung ultrasound study. Cardiovasc Ultrasound.

[30] Gargani L (2011). Lung ultrasound: a new tool for the cardiologist. Cardiovasc Ultrasound.

[31] Bouhemad B, Liu ZH, Arbelot C (2010). Ultrasound assessment of antibiotic-induced pulmonary reaeration in ventilator-associated pneumonia. Crit Care Med.

[32] Buddee FW, Johnson DC, Jellins J (1969). Experimental and clinical experiences in the use of ultrasound for the early detection of pulmonary emboli: a preliminary report. Med J Aust.

[33] Crawford HD, Wild JJ, Wolf PI, Finks JS (1959). Transmission of ultrasound through living human thorax. Ire Trans Med Electron.

[34] Lichtenstein DA (2015). BLUE-protocol and FALLS-protocol: two applications of lung ultrasound in the critically ill. Chest.

[35] Weinberger SE, Drazen JM, Kasper DL, Braunwald E, Fauci AS, Hauser SL, Longo DL, Jameson JL (2005). Diagnostic procedures in respiratory diseases. Harrison’s principles of internal medicine.

[36] Mayo PH, Beaulieu Y, Doelken P (2009). American College of Chest Physicians/La Société de Réanimation de Langue Française statement on competence in critical care ultrasonography. Chest.

[37] Lichtenstein D, Axler O (1993). Intensive use of general ultrasound in the intensive care unit. Prospective study of 150 consecutive patients. Intensive Care Med.

[38] Havelock T, Teoh R, Laws D (2010). Pleural procedures and thoracic ultrasound: British Thoracic Society Pleural Disease Guideline 2010. Thorax.

[39] Kiefl D, Eisenmann S, Michels G (2020). German recommendations on lung and thoracic ultrasonography in patients with COVID-19. Med Klin Intensivmed Notfmed.

[40] Michels G, Breitkreutz R, Pfister R (2014). Value of lung ultrasound in emergency and intensive care medicine. Dtsch Med Wochenschr.

[41] Gardelli G, Feletti F, Nanni A (2012). Chest ultrasonography in the ICU. Respir Care.

[42] Lichtenstein DA (2014). Lung ultrasound in the critically ill. Ann Intensive Care.

[43] Lichtenstein DA (2017). Lung ultrasound (in the critically ill) superior to CT: the example of lung sliding. Korean J Crit Care Med.

[44] Armbruster W, Eichholz R, Notheisen T (2019). Lung ultrasound for anesthesia, intensive care and emergency medicine. Anasthesiol Intensivmed Notfallmed Schmerzther.

[45] Efremov SM, Kuzkov VV, Fot EV (2020). Lung ultrasonography and cardiac surgery: a narrative review. J Cardiothorac Vasc Anesth.

[46] Ziskin MC, Thickman DI, Goldenberg NJ, Lapayowker MS, Becker JM (1982) The comet tail artifact. J Ultrasound Med 1:1–7 10.7863/jum.1982.1.1.1 6152918

[47] Schmid M, Escher F, Clevert DA (2020). Lung ultrasonography in COVID-19 pneumonia. Radiologe.

[48] Dietrich CF, Mathis G, Blaivas M (2016). Lung B-line artefacts and their use. J Thorac Dis.

[49] Lesser TG (2017). Significance of thoracic and lung ultrasound in thoracic surgery. Ultraschall Med.

[50] Goffi A, Kruisselbrink R, Volpicelli G (2018). The sound of air: point-of care lung ultrasound in perioperative medicine. Can J Anesth.

[51] Piscaglia F, Stefanini F, Cantisani V (2020). Benefits, open questions and challenges of the use of Ultrasound in the COVID-19 pandemic era The views of a panel of worldwide international experts. Ultraschall Med.

[52] Johri AM, Galen B, Kirkpatrick JN (2020). ASE statement on point-of-care ultrasound during the 2019 novel coronavirus pandemic. J Am Soc Echocardiogr.

[53] Guidelines for cleaning and preparing external- and internal-use ultrasound probes between patients, safe handling, and use of ultrasound coupling gel. American Institute of Ultrasound in Medicine website. https://www.aium.org/accreditation/Guidelines_Cleaning_Preparing. Last accessed 4 March 2021 10.1002/jum.16167 36655607

[54] ACEP Guideline on COVID-19 (2020). Ultrasound machine and transducer cleaning. Ann Emerg Med.

[55] Nyhsen C, Humphreys H, Koerner R (2017). Infection prevention and control in ultrasound—best practice recommendations from the European Society of Radiology Ultrasound Working Group. Insights Imaging.

[56] Ai A, Anderson L, Safdar N (2020). Barriers and facilitators to standardization of ultrasound use and probe disinfection in the ambulatory setting. Infect Control Hosp Epidemiol.

[57] Lu W, Zhang S, Chen B (2020). A clinical study of noninvasive assessment of lung lesions in patients with coronavirus disease-19 (COVID-19) by bedside ultrasound. Ultraschall Med.

[58] Soummer A, Perbet S, Brisson H (2012). Ultrasound assessment of lung aeration loss during a successful weaning trial predicts postextubation distress. Crit Care Med.

[59] Bai HX, Hsieh B, Xiong Z (2020). Performance of radiologists in differentiating COVID-19 from viral pneumonia on chest CT. Radiology.

[60] The Division of Perinatology, Society of Pediatric, Chinese Medical Association et al (2019) New guidelines for ultrasonic diagnosis of neonatal lung diseases. Chin J Contemp Pediatr 21(02):105–113

[61] Leech M, Bissett B, Kot M (2015). Lung ultrasound for critical care physiotherapists: a narrative review. Physiother Res Int.

[62] Huang Y, Wang S, Liu Y et al (2021) A preliminary study on the ultrasonic manifestations of peripulmonary lesions of non-critical novel coronavirus pneumonia (COVID-19). https://papers.ssrn.com/sol3/papers.cfm?abstract_id=3544750. Last accessed 4 March 2021

[63] Blaivas M, DeBehnke D, Phelan MB (2000). Potential errors in the diagnosis of pericardial effusion on trauma ultrasound for penetrating injuries. Acad Emerg Med.

[64] Clevert, Schröder, Sabel, Atemnot und Ultraschall, Bayerisches Ärzteblatt 5/2020 S. 204

